# Drill wobble – effect on femoral tunnel aperture during anterior cruciate ligament reconstruction

**DOI:** 10.1186/s40634-016-0073-1

**Published:** 2016-12-12

**Authors:** Naser Alnusif, Adam Hart, Maher Baroudi, Robert Marien, Mark Burman, Paul A. Martineau

**Affiliations:** Division of Orthopaedic Surgery, McGill University Health Centre, 1650 Cedar Avenue, A5-175.1, Montreal, QC H3G 1A4 Canada

**Keywords:** Anterior cruciate ligament, Femoral tunnel placement, Wobble effect, Anterior cruciate ligament reconstruction failure

## Abstract

**Background:**

In anterior cruciate ligament reconstruction performed using cortical button fixation on the femur, we have observed a “wobble” effect that can occur when a cannulated femoral drill is used over a guide pin that is not securely fixed in bone. Our study assessed the effect of drill “wobble” on femoral tunnel aperture in sawbones.

**Methods:**

Femoral tunnels were drilled in sawbones, which had been divided in two groups of 10 each, per drilling technique. The “wobble” technique group had the smaller cortical button drill passed before drilling the graft socket with the bigger diameter femoral drill. In contrast, in the “non-wobble” technique group, the smaller cortical button drill was passed after drilling the graft socket. The aperture dimensions: antero-posterior, proximo-distal and oblique, as well as the length of each tunnel, were measured.

**Results:**

While the average dimensions of the tunnels were similar between the two techniques, there was significantly more variation in the antero-posterior measurements for the wobble technique as compared to the non-wobble technique (mean 7.3 mm, SD 0.28 mm, and mean 7.3 mm, SD 0.11 mm, respectively; Brown-Forsythe test, *p* 0.02).

**Conclusion:**

We conclude that using the “socket first” “non-wobble” technique is a single surgical technical step surgeons can employ to decrease variability in tunnel aperture and size.

## Background

There are numerous technical options to choose from when reconstructing the ACL such as: (Hensler et al. 2011; Kato et al. 2010; Zantop et al. 2008a) anatomical versus isometric placement of the tunnels, type of graft, type of implant to secure the graft and various drilling techniques. While femoral tunnel position is a well-known determinant of successful reconstruction (Carson et al. 2004; Diamantopoulos et al. 2008; Hosseini et al. 2012; Kato et al. 2010; Moon et al. 2014; Niki et al. 2015; Sommer et al. 2000; Stevenson and Johnson 2007; Zantop et al. 2008a), the shape and aperture of the tunnel are also important factors to take into consideration (Hensler et al. 2011) especially when aiming for an anatomical tunnel placement.

In cases where a cortical button is employed to secure the graft to the femur, our group has observed an unsteady movement of the guide pin that is not securely fixed in bone when over drilled with a cannulated femoral drill. This common scenario is presumed to occur when the drilling sequence produces the smaller cortical button drill hole before the larger graft tunnel socket. In this situation, the tunnel guide pin would be loose within the cortical button drill hole. As a consequence of drilling over a loose guide pin, the drill might “wobble” and possibly alter the tunnel shape and aperture dimensions. To our knowledge, this observation was not reported in the literature with no previous study comparing the effect of changing the drilling sequence of the femoral tunnel on the tunnel shape.

Given the importance of femoral tunnel morphology, our study assessed the effect of “drill wobble” on femoral tunnel aperture in an artificial bone model. Our primary outcome was to compare the mean and variance in tunnel aperture dimensions at the bone surface in specimens drilled with and without drill wobble. We hypothesized that drill wobble would produce less consistent tunnel aperture dimensions.

## Methods

**Fig. 1:**
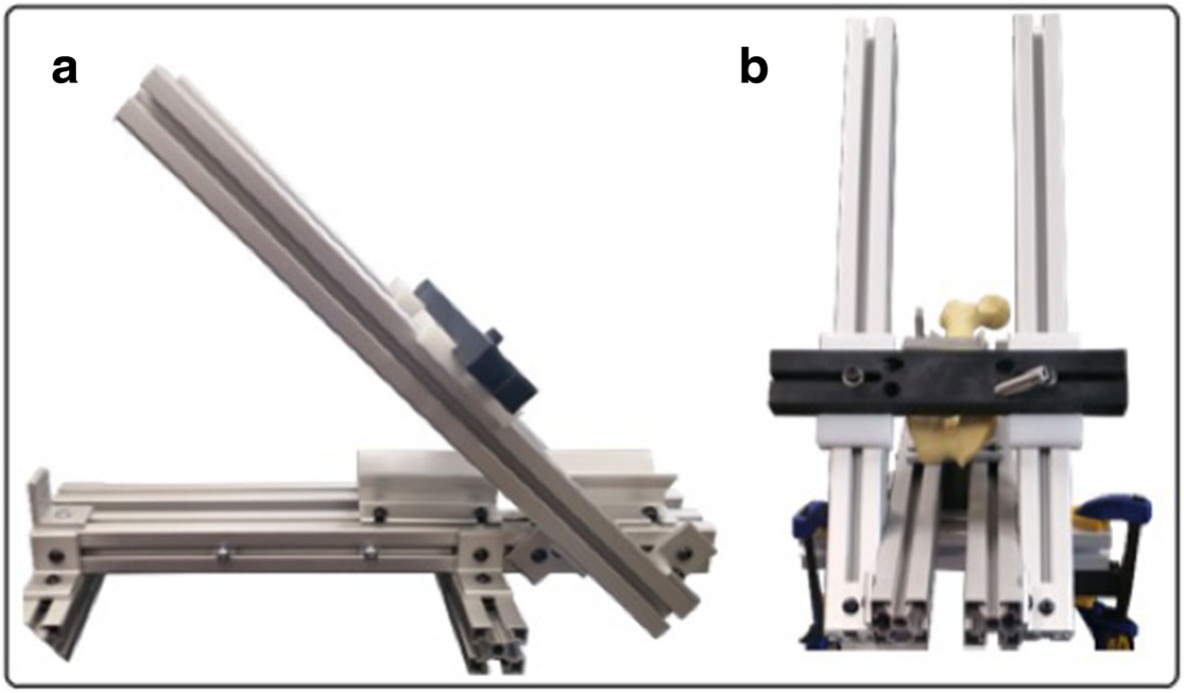
Custom made drill guide with fixed angles to standardize the drilling techniques (**a**) Side view with 45° fixed angle (**b**) Front view with a Sawbone sample fixed in the guide

**Fig 2:**
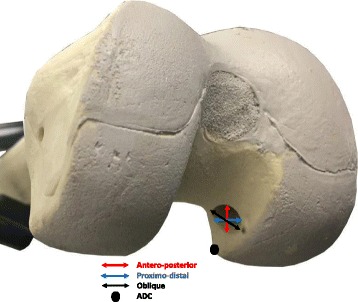
Painted distal femur articular cartilage with the tunnel dimensions and ADC marked

Twenty left solid foam artificial femurs measuring 42 cm in length and an interepicondylar diameter of 8.5 cm (Sawbones Model #1120, Pacific Research Laboratories, Inc., Vashon, WA, USA) were used and divided into two equal groups. We elected to use artificial bones to avoid inter-specimen variability as well as the effect of different age and suspected bone quality inherent to cadaveric studies. Similar artificial bones where used successfully in previous studies (Hamilton et al. 2011; Zantop et al. 2008b). A custom drill guide was used to standardize the drilling with fixed angles (Fig. 1), 45° from superior to inferior in the sagittal plane and 45° from medial to lateral in the coronal plane (Fig. 1a & b). Angles were used following the standard anteromedial portal technique based on the study performed by Moon DK et al. (Moon et al. 2014) to avoid extreme angles, blowout, and damage to the medial femoral condyle. Hensler et al. also concluded that a transverse drill angle of 40° resulted in an aperture that is closest to the native ACL footprint. The femurs were clamped parallel to the floor onto the custom jig to reproduce the exact anatomical position and minimize variability between specimens. A 2.4 mm guide pin was then placed in the center of the ACL femoral footprint on each specimen, which was located 3 mm anterior and 12 mm distal to the apex of the deep cartilage (ADC) as described by Hart et al. (Hart et al. 2015). Using a clear dot that represented the ADC in all identical sawbones (Fig. 2), the entry point was marked prior to drilling.

In the “wobble Technique” group, the cortical button drill bit of 4.5 mm (Acufex femoral drill, Smith & Nephew, Andover, MA, USA) was initially drilled over the guide pin all the way through the lateral femoral cortex. Then a larger drill bit of 7 mm diameter (Acufex femoral drill, Smith & Nephew Andover, MA, USA) was used to create the graft socket. While in the “non-wobble technique” group, the graft socket was first drilled using a 7 mm drill bit over the guide pin. Then the 4.5 mm cortical button drill bit was used to drill all the way through the lateral femoral cortex. All samples were drilled by a single sports fellowship trained surgeon.

Once all the models were drilled, a digital calliper (Empire 6 in. Digital calliper, Model#2789, Mukwonago, WI, USA) was used to measure four dimensions of the tunnel entry point: antero-posterior, proximo-distal, oblique (antero-proximal to postero-distal), and tunnel length (Fig. 2). Measurements were rounded to the nearest 1/10^th^ of a mm and were taken by the first author (N.A.) to eliminate the inter-rater reliability and given the accuracy of the digital caliper to 0.0005/0.25 mm and the fact that the measurements were simple linear distances would decrease the chance of intra-rater errors.

### Statistical analysis

Tunnel aperture measurements are summarized and reported as means and standard deviations. Separately for each dimension of measurement, we compared means using a *t*-test for independent samples and variances using a Brown-Forsythe (BF) test for independent samples (Brown and Forsythe 1974), between the non-wobble and wobble techniques. Results for the comparison of means are reported as difference of means and 95% confidence interval (CI) for the difference, as well, as the test statistic t, degrees of freedom (df) and *p* value, for the *t* test. On the comparison of variances, we reported the variance (to two decimals (Altman et al. 1992)), the ratio of the two variances and 95% (CI) for the ratio (the null value for the ratio is 1), as well, as the test statistic F, numerator and denominator df and *p* value, for the BF test.

All statistical tests of hypothesis were two-sided and performed at the significance level of 0.05. All statistical analyses were done using the SAS software, version 9.3 (SAS Institute, Inc., Cary, NC, USA). Power analysis mandated at least 8 samples per group in order to detect a difference in tunnel aperture of 1 mm with power of 0.8 assuming a typical tunnel diameter of 7 mm +/− 0.5 mm (Kane SP Clincalc.com. http://clincalc.com/Stats/SampleSize.aspx).

## Results

Table 1 shows the means and standard deviations for the different dimensions (antero-posterior, proximo-distal, oblique and length) of the aperture measurements of the non-wobble and wobble techniques. Results of the *t*-tests and Brown-Forsythe tests performed are also reported.

**Table 1:** Descriptive statistics for tunnel aperture measurements and comparison of means and variances between the wobble and non-wobble technique (*n* = 10 per group)

				*t* test		Brown-Forsythe test	
Dimension	Technique	Mean	SD	*t* ^a^	*p* value	*F* ^b^	*p* value
Antero-posterior	NW	7.3	0.1	−0.01	0.9	6.89	0.02^c^
	W	7.3	0.3				
Proximo-distal	NW	7.7	0.1	1.91	0.1	1.61	0.2
	W	7.6	0.3				
Oblique	NW	8.1	0.2	0.76	0.5	0.06	0.8
	W	8.1	0.2				
Length	NW	27.2	0.8	−2.05	0.1	0.01	0.9
	W	28.0	0.9				

*NW* non-wobble, *W* wobble, *SD* standard deviation

^a^Degrees of freedom for *t* = 18

^b^(Numerator, Denominator) degrees of freedom for *F* = (1, 18)

^c^statistically significant

The comparison of mean tunnel aperture measurements between the two techniques showed no statistical significant difference in any of the different dimensions studied. For the antero-posterior measurements, the mean difference (defined as non-wobble minus wobble) was 0 mm (95% CI (−0.2, 0.2)); for the proximo-distal, the difference was 0.2 mm (95% CI (−0.02, 0.4)); for the oblique it was 0.1 mm (95% CI (−0.1, 0.3)) and for the length it was −0.8 mm (95% CI (−1.6, 0.02)).

The comparison of variances showed no statistical significant difference for the dimensions proximo-distal, oblique and length. For proximo-distal, the variances where 0.02 and 0.07 for the non-wobble and wobble techniques, respectively (ratio 0.3, 95% CI (0.1, 1.3)). For oblique, the variances were both 0.06 (ratio 1.0, 95% CI (0.2, 4.0)). For length, the variances were 0.71 and 0.79 for the non-wobble and wobble techniques, respectively (ratio 0.9, 95% CI (0.2, 3.6)).

There was, however, a statistical significant difference between the variances of antero-posterior measurements. The variances were 0.01 and 0.08 for the non-wobble and wobble techniques, respectively, for a ratio of 0.2 (95% CI (0.04, 0.7); BF test, *p* 0.02).

## Discussion

**Fig. 3:**
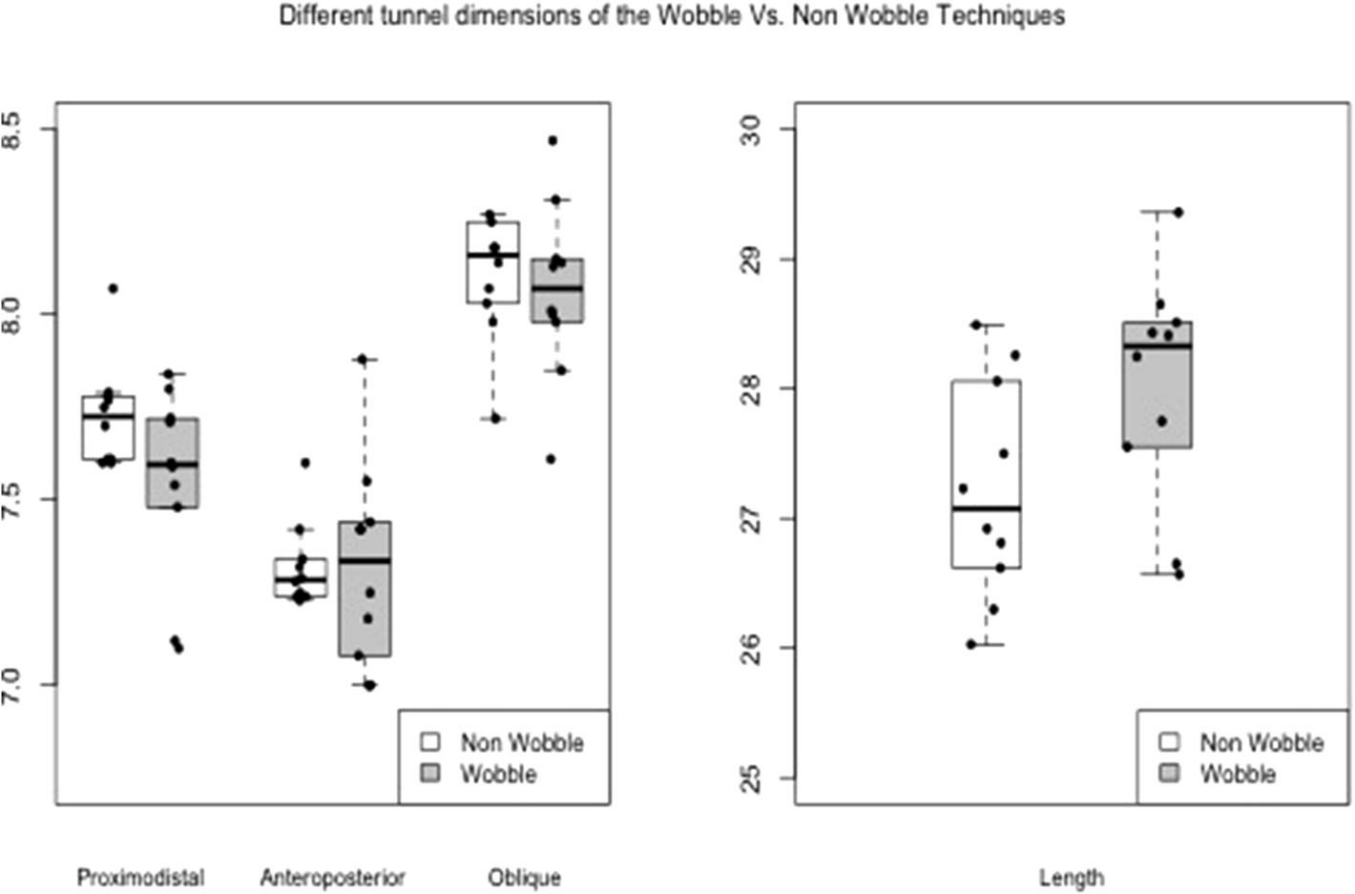
Boxplots showing the distribution of tunnel aperture measurements of the 4 different dimensions between the wobble & non-wobble techniques, with the apparent higher variability for the wobble technique in the antero-posterior dimension

While there has been extensive research on ACL tunnel positioning with various reconstruction techniques (Bedi et al. 2010; Chen and Wang 2015; Heming et al. 2007; Lim et al. 2012; Mayr et al. 2016; Musahl et al. 2005; Tiamklang et al. 2012; Yagi et al. 2002; Zavras et al. 2005), the effect of a single surgical technical modification of the order of femoral drilling technique on tunnel aperture has not been described. In this study, the effect of drilling over a loose guidewire confirms the presence of a wobble effect. Specifically, sawbones that were drilled in the wobble technique yielded higher variation in the antero-posterior dimensions compared to the non-wobble technique (Fig. 3); confirming our hypothesis that the wobble effect exists. As a consequence, surgeons using the wobble technique should be aware that femoral tunnel dimensions and aperture are less consistent when using this drilling sequence.

While multiple studies emphasize the importance of anatomic tunnel placement (Hensler et al. 2011; Moon et al. 2014; Zantop et al. 2008a), Hensler et al. elaborated extensively on the femoral tunnel footprint with the effect of drill size, transverse drill angle and to a lesser extent knee flexion angles on femoral tunnel aperture geometry. In their literature search, the average femoral insertion site was 8.9 mm wide, which corresponds to the oblique dimensions in our study , however they did not assess the effect of different drilling sequence on the femoral tunnel footprint which from our study we found that this also has a significant effect on the tunnel aperture geometry.

There was no significant difference in total tunnel length between the two groups with non-wobble technique (Mean = 27.2, SD = 0.8) compared to the wobble technique (Mean = 28.0, SD = 0.9) P = 0.9. Furthermore, both groups tunnel lengths fall into the presumed safe minimum tunnel length of 25 mm for adequate bone tendon integration (Bedi et al. 2010; Chang et al. 2010; Golish et al. 2007), which is thought to decrease failure rate. Furthermore, Hensler et al. (Hensler et al. 2013) performed a review of the literature and found no strong evidence that supports the theory of minimal limit of 25 mm tunnel length for bone-tendon healing. When comparing the proximo-distal, antero-posterior and oblique dimensions between the two groups, we found no significant difference in the means between groups.

With regards to the variance however, the wobble effect did produce significantly more variable tunnels in the antero-posterior dimension. When aiming for an anatomical ACL reconstruction, increased variability in tunnel aperture might be undesirable as it may contribute to inconsistent clinical outcomes and potentially higher complication rates due to failure to reproduce the native ACL footprint as well as increasing the chance of posterior wall blowout. In theory, the increased variability of the tunnel dimensions that results from the wobble effect may change the center of the tunnel location potentially contributing to graft malpositioning , which could alter the biomechanics of the knee and potentially lead to instability and graft failure (Hensler et al. 2011; Jepsen et al. 2007; Kopf et al. 2010). However, our study mainly assesses the effect of the two different drilling techniques on tunnel geometry and clinical significance is unknown so further clinical studies comparing both drilling techniques need to be performed to quantify the clinical importance of the wobble effect.

The wobble effect can be worsened in certain clinical scenarios such as having a bigger graft size thereby requiring a bigger diameter femoral drill than the one applied in our study. The use of a larger femoral drill could compound the results we demonstrated experimentally with the use of the 7 mm drill. Therefore, using larger femoral drills could increase the range and variability and theoretically increase the risk of posterior wall blowout. This theory is supported by Hester et al. (Hensler et al. 2011) who described the strong impact small changes in drill-bit diameter on tunnel aperture size and morphology. The same concept applies to other clinical scenarios like dealing with a narrow and or deep femoral notch which would alter the femoral drilling angle requiring even more tangential drilling, once again compounding the potential range and variability of aperture dimensions and increasing the risk of injuring the medial femoral condyle and cartilage (Wang et al. 2012). However, further work is necessary to quantify the effect of these variables on the wobble effect.

From the results of this study, a change in one step in the technique used in ACL reconstruction by drilling the larger femoral graft socket over the guide pin prior to using the cortical button drill bit leads to less variability in tunnel aperture and geometry. .

The main limitation in our study was the use of sawbones which may differ from true human anatomy with the surrounding soft tissues that might affect the drilling technique. Nonetheless, standardizing the drilling technique and specimens with the drill guide assured reproducibility of the results. It is unlikely the aperture measurements would have been different in real bone.

## Conclusion

In ACL reconstruction, where surgeons seemingly strive to reproduce the native anatomy to the closest millimeter and the literature is filled with studies describing ACL footprint characteristics to the nearest detail, it is surprising that we previously had no information about how the simple sequence of drilling could affect tunnel aperture characteristics. We conclude that using the “socket first” “non-wobble” technique is a single surgical technical step surgeons can employ to decrease variability in tunnel aperture and size.

## Abbreviations

ACL: Anterior cruciate ligament
ADC: Apex of the deep cartilage
BF: Brown-Forsythe
CI: Confidence interval
DF: Degrees of freedom
SD: Standard deviation
